# Mapping do-it-yourself science

**DOI:** 10.1186/s40504-018-0090-1

**Published:** 2019-01-14

**Authors:** Federico Ferretti

**Affiliations:** Joint Research Centre of the European Commission, BLD 45, Office 119, Via E. Fermi, 2479, 21027 Ispra, VA Italy

**Keywords:** DIY science, Social worlds/arenas theory, The maker movement

## Abstract

The emergence of Do-It-Yourself (DIY) science movements is becoming a topic widely discussed in academia and policy, as well as by the general public and the media. While DIY approaches enjoy increasing diffusion even in official research, different social actors frequently talk about them in different ways and circumstances. Interaction and negotiation processes amongst actors (e.g. policy makers and DIY communities) define the premises upon which different conceptualisations of DIY science are deployed.

In this paper we offer a framework for analysing the discourse on DIY science.

Our study consists of a field research of three spaces active in DIY science premises, two dedicated events of the DIY community, and an auto-ethnography in the field of DIY biology.

By relying on the theory of social worlds/arenas (SW/A), we collected data on how notions of DIY science are constructed by different social actors and how conceptual borders are built or are likely to shift, resulting in multiple possible SW/A mappings. We conclude that each and every conceptualisation of DIY science cannot stand independently from the situatedness of its multiple contexts, therefore making its usage in policy making and governance particularly complex.

## Introduction

DIY science is gaining a great deal of attention both among practitioners and in the media. The locations of DIY science are various and range from small groups of tech enthusiasts to large online communities that share scientific objectives and outcomes, organised into local makerspaces, Fablabs, classrooms, universities and museums, public libraries and private enterprises (Nascimento, Guimarães Pereira, & Ghezzi, 2014). Such heterogeneity does not only result in different ways of practicing DIY science, but also in differences in its conceptualization.

Opportunities of DIY science typically gather a variety of social actors, making it difficult, if not impossible, to create fixed and unique categorizations about the phenomenon (Alper, 2013). Quite on the contrary, actors interact in many different ways, in different virtual or physical spaces, being moved by different beliefs, values, interests and visions, not least on the developments of DIY movements as such.

Our argument is that different social actors articulate discourse about DIY science in fundamentally different ways, pointing us to the existence of multiple understandings and, consequently, practical implementations of DIY science. To test this hypothesis, we direct our investigation to the level of social action where individuals share participation and commitment to DIY science, relying on the theory of social worlds/arenas (SW/A) to explore how “various issues are debated, negotiated, fought out, forced and manipulated by representatives” (Strauss, 1978, p. 124). Mapping SW/A renders visible these patterns of commitment and action and helps elucidating how discourse becomes performative in generating certain conceptualizations of DIY science against others.

While the origins of DIY can be traced back to the 60s and the 70s, the recent advent of the digital era and the Internet have drastically transformed the access to information and technologies, attracting greater audience to a number of newer sociotechnical issues, such as 3D print or genome editing, which often carry with themselves the promise of an extended participation to science. Such tendency has surely shortened the perceived distance between professional scientists and the general public,^1^ putting upfront questions on how such emerging social practices are going to affect social life at large, and, indirectly, value systems to think about science. As an example, CRISPR based technologies are offering once unprecedented opportunities for genome editing that frequently raise fervent reactions in the media: a 2015 Guardian piece entitled “Human gene editing is a social and political matter, not just a scientific one^2^” reminds us of the social and ethical dimensions brought about by the emergence of new technologies.

Yet, DIY science is directly and indirectly referred to with multiple understandings, beliefs, and expectations, entangling different views on themes such as innovation, ethics, new technologies, education, employment, and risk assessment.

This paper offers a framework to analyse the heterogeneity and complexity of DIY science by looking at how its actors articulate discourse about it. Following from such discourse analysis, we offer two, among many others, possible mappings of the social worlds and arenas in which DIY science is situated.

Our inquiry into DIY science projects, experiments, online and offline communities, and laboratories allowed us identifying (i) the main social actors populating DIY science (and consequently, their social worlds and arenas), and (ii) how the actors’ articulation of discourse may result in different mappings of DIY science.

We studied DIY science with a dual approach: first we conducted field research of three spaces across Europe active with DIY science projects and two technology and innovation international fairs where communities of DIY scientists and institutional representatives meet. Second, we engaged at the micro level with our local community by setting up a DIY science project in the field of biology.

Section two deals with the notion of DIY science and its origins, as well as with the heterogeneity and variety of the DIY settings. It also points out the substantial lack of studies on how the actors of the DIY panorama form and negotiate their perspectives, as well as organize their social life. Section three is an account of social world/arenas theory and how it is meaningful for our scope. Section four describes the methodology we adopted for our social research, made of an auto-ethnographic and an in-field research phases aimed at identifying the actors and their interactions and practices within different worlds and arenas. An analysis of such arenas is offered in section five, that puts the basis for further discussion even on some seemingly contradictory characteristics of DIY science.

## DIY science

The expression “do-it-yourself”, abbreviated “DIY”, emerged in North America after World War Two initially referring to the “making” and “crafting” associated with the spread of hobbysm as a social practice (Gelber, 1999). Few publications from that time^3^ testimony the association of DIY with individual well-being and self-production, with particular focus on the construction and repair of household objects using common hands tools.

From that perspective, DIY was at first identified with the physical activity of *making,* trait that we find again today with the spread of the Maker Movement (see e.g. Chen & Wu, 2017a). In relation to hobbies and leisure, Menninger (1942) described how frequently the physical activity of making “may not be unrelated to a similar type of satisfaction gained from athletic activities, hiking or dancing” (p.123) as well as that “in not a few activities, maximum satisfaction is gained from the participation of the individual with a group, [which is often] a prerequisite to carrying on the activity”(p.128). It thus seems that, in an early conceptualization, DIY allowed individuals and groups to freely initiate and conduct projects with a direct observation of their work and consequent satisfaction about their accomplishments.

Beyond self-taught manufacturing, the expression was progressively appropriated in the language of the counterculture of the 60s and 70s, this time also implying a moral critique to the formal education systems, and more generally the consumerist society (Smith, 2014). Hence, DIY became also a mean for protest together with the spread of grassroots political activism. Duncombe (1997) describes DIY as “a critique of the dominant mode of passive consumer culture and something far more important: the active creation of an alternative culture. DIY is not just complaining about what is, but actually doing something different” (p.117). At least to some degree, this understanding seems to resonate with “prefiguring and constructing alternatives to existing institutions” (Day, 2005, p.19).

In the 80s and 90s, the DIY culture evolved towards new possibilities of media production, made more accessible and less expensive with the advent of the digital era, offered (especially within the nascent punk, free party and rave cultures in the UK) feasible possibilities also in the arts and crafts, politics and not least, science (Lowndes, 2016).

The potential of DIY approaches begun to be considered in official science: few examples include the Cornell Lab of ornithology, active since 1994 in the gathering and elaboration of data on birds through extended participation and citizen engagement (see Trumbull, Bonney, Bascom, & Cabral, 2000), and the SET@home, launched in 1996 as an Internet distributed computing project for research purposes on interstellar space and extraterrestrial intelligent lifeforms^4^

Today, recent technological developments (among which mobile computing) are allowing even newer practices, unimaginable just two decades ago: a 2017 Nature article entitled “The DIY electronics transforming research” (Cressey, 2017) described how the impact of low cost microcontrollers and single board computers is changing the way research is nowadays conducted, in computer engineering particularly. Raspberry Pis, Arduinos and similar devices are nowadays commonly used in contexts such as gene editing, marine research, drug design and automation engineering, not least even by scientists from the academia: in 2017, 14 million of Raspberry Pis boards were sold worldwide.^5^ Such diffusion, Cressey argues, greatly reduces the amount of preliminary knowledge necessary for people normally external to the academia to conduct experiments, or in other words, to “do science”. Drawing from our collective imagination of Galileo and Newton, we are familiar with the figure of independent scientists mainly moved by a pure pursuit of knowledge and used to set up experiments to verify their theories of nature. Increasing consensus is forming that a similar curiosity-driven approach to research is coming back, albeit on other grounds, also thanks to the spread of DIY methods (Dance, 2017): expensive experiments that once required institutional or private founding are now replicable with a fraction of the resources, while, at the same time, peer communities still offer technical support and expertise (Kuznetsov & Paulos, 2010).

Still, what different actors more precisely mean when they refer to DIY science? Accounts on the meanings of DIY science are scarce in the literature. While Citizen Science has been described as a collective scientific *practice* (Cooper et al., 2014; Morzy, 2015; Newman et al., 2012), DIY science rather more broadly refers to the *process* initiated by individuals and groups that tinker, hack, fix, and recreate objects and systems out of their own interest, curiosity or need, and openly share results and outcomes in their networks (Nascimento, Guimarães Pereira and Ghezzi, 2014). Not only amateurs, tech-enthusiasts and hobbyists appear among the actors of DIY science, but also an increasing number of professional scientists, teachers and university professors (Haklay et al., 2018). Such constitutive heterogeneity makes difficult and arbitrary to outline the boundaries of DIY science. Much of the available information on the topic typically builds upon the analysis of instances of the Maker Movement as it frequently offers a physical space for DIY science projects (Austen, 2013). However, in order to identify the broad spectrum of DIY science manifestations, we cannot a priori exclude additional circles such as communities of practice, expert groups, online platforms, or simply, private garages. In this premises, additional actors act upon different concerns that typically entail different understandings of DIY science. For that reason, we designed our research study to be as inclusive as possible of such variety (see Perspectives on DIY science section).

### Literature study

We additionally performed a search on Scopus^6^ to assess and confront meanings of DIY science in the academic literature and check the thematic angles from which DIY science is tackled. After reviewing a pool of 29 papers, we classified them according to their disciplinary angle and main area of interest. The papers are summarized in the following table (Tables 1 and 2).

**Table 1:** Selected papers on DIY science

Reference	Title	Year	Main area(s) of interest
Chen & Wu, 2017	The hot spot transformation in the research evolution of maker	2017	The Maker movement (review paper)
Tofel-Grehl et al., 2017	Electrifying Engagement in Middle School Science Class: Improving Student Interest Through E-textiles	2017	The Maker Movement and education
Wexler, 2017	The social context of “do-it-yourself” brain stimulation: Neurohackers, biohackers, and lifehackers	2017	Neurohacking and ethics
Lehr et al., 2017	Communicating landscape hydrology — the water cycle in a box	2017	DIY approach to hydrological modelling
Berditchevskaia, Regalado, & Duin, 2017	The changing face of expertise and the need for knowledge transfer	2017	DIY knowledge production and transfer
Brown, 2017	If you want something doing, do it yourself	2017	Democratization of technology, quality control
Brown et al., 2017	Evolving skills for emerging technologies: a collaborative approach	2017	Skill development in the context of archival studies
Vandevelde, Wyffels, Ciocci, Vanderborght, & Saldien, 2016	Design and evaluation of a DIY construction system for educational robot kits	2016	Educational robotics
Lamore, 2016	Fan cart: The next generation	2016	DIY and engineering skills in education
Sleator, 2016b	Synthetic biology: from mainstream to counterculture	2016	Synthetic DIY Biology
Sleator, 2016a	Diy biology-hacking goes viral!	2016	Emergence of DIY as a social phenomenon
Nguyen, 2016	Make magazine and the social reproduction of DIY science and technology	2016	The Maker Movement
Bardaji, Sánchez, Simon, Wernand, & Piera, 2016	Estimating the underwater diffuse attenuation coefficient with a low-cost instrument: The KdUINO DIY buoy	2016	DIY method for water testing, Citizen Science implications
Richards, 2016	Shifting Gender in Electronic Music: DIY and Maker Communities	2016	The Maker Movement and ethics
Busch et al., 2016	Citizen bio-optical observations from coast- and ocean and their compatibility with ocean colour satellite measurements	2016	DIY method for Citizen Science in the context of marine science
Fourie & Meyer, 2015	What to make of makerspaces: Tools and DIY only or is there an interconnected information resources space?	2015	The potential of the Maker Movement in public libraries
Davies, Tybjerg, Whiteley, & Söderqvist, 2015	Co-Curation as Hacking: Biohackers in Copenhagen’s Medical Museion	2015	DIY bio in the context of museums
Eggleson, 2014	Transatlantic Divergences in Citizen Science Ethics—Comparative Analysis of the DIYbio Code of Ethics Drafts of 2011	2014	DIY bio and ethical aspects
Yang, Patsavas, Byrne, & Ma, 2014	Seawater pH measurements in the field: A DIY photometer with 0.01 unit pH accuracy	2014	DIY prototyping of spectrophotometric systems
Seyfried, Pei, & Schmidt, 2014	European do-it-yourself (DIY) biology: Beyond the hope, hype and horror	2014	DIYbio in European Citizen Science
Mereu & Villarroel, 2014	Visions Project K.1: DIY 3-D interactive videohologram device	2014	Prototyping of a 3D video application
Fortunati, Esposito, Ferrin, & Viel, 2014	Approaching Social Robots Through Playfulness and Doing-It-Yourself: Children in Action	2014	Learning by doing in school
Strickland, 2014	Brain hacking: Self-experimenters are zapping their heads	2014	Neurohacking and ethics
Landrain, Meyer, Perez, & Sussan, 2013	Do-it-yourself biology: Challenges and promises for an open science and technology movement	2013	DIYbio and ethics
Delgado, 2013	DIYbio: Making things and making futures	2013	DIYbio vs instutionalized biology
Buechley & Perner-Wilson, 2012	Crafting technology: Reimagining the processes, materials, and cultures of electronics	2012	Survey study on DIY electronics practices
Rennie, Evans, Mayne, & Rennie, 2010	Factors affecting the use and outcomes of interactive science exhibits in community settings	2010	Citizen Engagment in science
Kelty, 2010	Outlaw, hackers, victorian amateurs: Diagnosing public participation in the life sciences today	2010	Citizen Engagment in science
Wan, Wu, & Chen, 2007	Application of program generation technology in solving heat and flow problems	2007	DIY approaches in thermal science

In approximately a quarter of all papers DIY science is tackled from the perspective of the Maker Movement and, in fewer cases, of Citizen Science. These contributions examine the broader context of DIY and maker practices by assessing how these latest enrich and diversify technology development. Therein, the Maker Movement is typically framed as a transformation “changing innovation, culture and education not only through open Internet technology and digital things, but also through physical things such as hardware designs, sensors, and networking devices” (Lindtner et al., 2014 p. 147 in Chen and Wu, 2017a, b). Another eight papers report on real case examples where DIY methods have been employed for the set up and conduction of content specific experiments. Despite being mostly technical, in more than one case few considerations are drawn about the DIY paradigm and its potential in science experiments. In six other papers DIY science is conceptualized in the context of biology (frequently through the expression “DIY Bio”) whereby “a new era of DIY Biology originally evolved as a non-institutional pursuit, with practitioners – many of whom having little or no formal training either due to a lack of infrastructure, funding or indeed opportunity – operating out of garages or modified kitchens” (Sleator, 2016a, b p.278). Amateur biologists have also been defined as “individuals who conduct biological experiments as an avocation rather than a vocation”(National Science Advisory Board for ger, 2011). All retrieved articles on the topic are concerned with reviewing the emergence and characteristics of the phenomenon, in some cases reporting upon few real case examples. Other common aspects of investigation are the implications of DIY methods in learning and skills development,^7^ and, to a lesser extent, the ethical consequences of the rise of DIY approaches and perspectives on the study of public participation to science.

### Perspectives on DIY science

Together with the topic of DIY science reverberating from different thematic and disciplinary angles, increasing literature reports on the growing suspicion with which science experts are being regarded by the general public (Gauchat, 2012; Lave, 2012; Wynne, 2007). As trust in mainstream science deteriorates, “individuals begin to give more weight to personal accounts and information shared within networks of peers” (Berditchevskaia et al., 2017, p. 1). On their side, scientists themselves are beginning to realise the scientific relevance of DIY practices,^8^ and examples of engagement in scientific research and dissemination are becoming more frequent outside the academia, either in the private sector or in own business (Cressey, 2012; Fritsch & Krabel, 2012). The DIY paradigm welcomes anyone to get involved and perform own research without the need for formal qualifications, and at the same time, it also enjoys good reputation within the scientific community (Wylie et al., 2014). As these practices become more integrated into the formal science system, scientists themselves are determined to improve and refine them in the light of their internal challenges (Garbarino & Mason, 2016). As a result, alternative scientific approaches are increasingly looked at by institutions and the academia not just benevolently, but also with indirect expectations about the scientific value that DIY science can lend to societal challenges (Lindtner & Lin, 2017). References to DIY science are not rare in policy making^9^ and the academia, with more and more frequent cases of DIY lab set up by researchers to contrast the bureaucracy and rigidity of universities^10^.

However, since DIY communities are not moved by a single goal, misconceptions about DIY science are around the corner: for instance, while amateur biology movements worldwide have shown a huge variety of research interests (Seyfrie et al., 2014), universities and policymakers are rather focused on a narrow definition and regulation of the ‘connected risks’ (Nature, 2013). Controversies arise widely when ethical issues become evident in the debate: should open genomics be regulated? What are the moral aspects of synthetic biology (see e.g. James, 2015)?

In some cases, these topics become matter of interest in the public sphere: a UNESCO panel of experts called for a temporary ban on genetic editing of the human germline, aiming for a wide public debate on genetic modification of human DNA.^11^ Interestingly, this debate started outside mainstream institutions, when one of the most important organisations in the promotion of DIY science in Europe, the Waag society in Amsterdam, initiated a public discussion about the legitimacy of CRIPSR toolkits.^12^

## Social worlds/arenas theory

According to Clarke (2003), new methods are needed to empirically investigate the increasing complexity and heterogeneity of modern societies. In a ‘postmodern’ era, she argues, *all* knowledges are socially and culturally produced and, for that reason, they need to be conceptualised as *situated*, i.e. produced and consumed by particular groups of people, “historically and geographically locatable” (p. xxv). The etymology of ‘situation’ has roots in the Latin *situatio*, literally “place, position, or location”^13^ and reminds of the physical connotation of the term. However, other than the temporal and spatial dimensions, Clarke asserts that situations can have multiple other orderings such as technological, work, sentimental, moral, and aesthetic, and that the units of analysis are the collective commitments and actions taken by the participants of a certain organisation (citing Strauss, 1978). For that reason, the analytical focus needs to go beyond the sole domain of social action, somehow integrating a broader perspective on elements of negotiation and discourse.

Social worlds and arenas can be conceptualised as a merely “extended situation” (Clarke, 2003, p.126) whose comprehension requires looking “at their embeddedness in a larger negotiated order” as “one cannot understand a social world in isolation” (Clarke, 2003, p. 138). In social worlds, groups of actors and individuals share commitments, activities, resources, goals and sometime build ideologies that can be explored by focusing on how issues are debated, negotiated, fought out, forced and manipulated (Strauss, 1978).

The literature is rich in examples of SW/A theory applied as a framework to discuss negotiations, conflicts and ideologies in health policy (e.g. Garrety, 1998; Karlberg, 2000; Neilson et al., 2013), cultural studies (e.g. Salin & Pesso, 2017), knowledge production and applied research (e.g. Andersen, 2014; Lin, 2011), business (e.g. Vasconcelos, 2007), etc.

### SW/a and DIY science

In section 2 we have illustrated how DIY science is regarded in the literature from a variety of viewpoints that sketch out its multiple faceted characteristics sometimes made of different relations among individuals, the collective, organisations and institutions. We believe that the theory of SW/A can offer a solid methodology to analyse DIY science’s discourse material, irregularities, contestations, contradictions and fragmentations. We illustrate possible understandings of DIY science using maps. Clarke (2003) discusses the usefulness of analysing situations using maps as they “open up knowledge spaces, [...] are great boundary objects-devices for handling multiplicity and heterogeneity…” and “are excellent devices to materialize questions” (p.30). Therefore, in order to assess the usefulness of mapping SW/A in the context of DIY science, we looked out for collective commitments, relations, and sites of action within the phenomenon.

In our context, social worlds become dedicated physical and virtual spaces such as makerspaces, Fablab and online communities, platforms and blogs and not least, events and occasions of interface among DIY scientists and institutions (e.g. conferences, university events, and sponsored tech-fairs). In these worlds we sought for how groups and individuals interact with distinct sets of norms, values, beliefs, communication styles, standards and language. In the attempt to shed light on the actors’ involvement in their social worlds, we departed from the actors’ proximity to the activities of their social worlds and the knowledge of their functioning.

## Methodology

In order to investigate DIY science and its involvement in different social worlds and arenas we designed a dual approach made of a field research phase and an auto-ethnographic phase. The first, which consisted of in person visits and participation to some activities typical of the DIY science scenario, revealed fundamental in informing the second phase, which focused instead on our personal involvement in a DIY biology project about the assessment of food quality. While the principal scope of the first phase was the identification of the social worlds of DIY science and their network placement, the second phase allowed in depth reflections on first hand practices and interactions in a particular project setting.

### Phase 1 –field research of the DIY science arenas and worlds

The main scope of the first phase was to probe the DIY science arenas and identify its main actors and groups, which we later catalogued in worlds and sub-worlds according to the theoretical framework of SW/A.

We visited three spaces across Europe: two Fablabs^14^ in Switzerland and Italy and one collaborative space in the Netherlands over different periods in 2016 to 2017. We actively took part to the spaces’ daily routine and directly observed DIY scientists work practices and their inter-relations and collaborations. Visits lasted a total of 6 working days (Table 2).

The three spaces feature quite different organisational styles:

**Table 2:** Visited spaces

#	Characteristics	Main focuses
Space 1	Located inside a university campus and therefore under the direct responsibility of the faculty. Small space, well equipped with 3d printing and other technologies for prototyping. Open to the faculty students only, under the direction of one senior member	Design, architecture, prototyping, workshops organization
Space 2	Big independent collaborative hub that includes sub-spaces (among which a Fablab and a DIY bio lab) that host activities of art, technology, and science. Open to everyone and ruled by a member board	DIY culture, collaborative economy, sustainability
Space 3	Supported by the local association of handicraft industry (national funds), particularly focused on providing assistance and tools for entrepreneurship. Additional on-demand services	3D printing, start-up business, prototyping

Additionally, we also attended two international events in Italy and France in 2016 over a total of 7 days. Such meetings gather the DIY community on themes such as innovation, science and the future, and are highly informative of the established communication networks (Table 3).

**Table 3:** Attended events

#	Characteristics	Main focus
Event 1	European reference event of the Maker Movement. Participants showcase their projects and engage among themselves and with the visitors.	Arts, crafts, engineering, science, DIY culture
Event 2	Principal event of an international NGO. Gathers innovators, entrepreneurs, tech enthusiasts and scientists around specific themes that are discussed in talks and plenary discussions.	Collaborative economy, creativity, fairness, openness and trust

During our field research, we wrote accurate descriptions of the observations and additionally carried out few open ended interviews with key actors in informal sessions around specific topics of discussion. We consulted conference programs and therein contained references to additional DIY activities and spaces, with the objective of compiling a basic social network map. Subsequently, we retrieved additional documents and material publicly online (organizations and associations websites) to enrich and confront our data. Typically, our observations revolve around how participants articulate discourse over the DIY movement, how they jointly organise work and establish communication within their group and towards the external.

### Phase 2 – Auto-ethnography of DIY bio in the field of food quality assessment

A 2014 report from European Commission mapped some among the most influential DIY projects across Europe (Nascimento et al., 2014, b, p. 42), showing that a good majority of them is concerned with environmental and climate related issues. The measurement and sensing of air and water quality are a common case within the DIY community, supported by many online sources that offer guidance on how to conduct experiments and share results (for a review see Kumar et al., 2015). Such a noticeable disciplinary preference made us curious to explore the coverage of DIY science in other scientific domains. We profited from our unique institutional closeness with different research groups at the Joint Research Centre of the European Commission (JRC) and went about choosing a subject that has enjoyed little attention from a DIY perspective: food quality. At the JRC food safety is an issue of major concern^15^ with research also motivated by a spread image of consumers reacting to food scares steadily changing their consumption styles according to their attitudes to risk (Van Rijswijk & Frewer, 2012; Yamoah & Yewson, 2014). Because recent food scams have also had echo in the media, which unanimously called for stronger food safety policies and harsher penalties,^16^ the JRC carries out a monthly initiative on food frauds and quality stating that consumers have the right to make informed decisions about the food they purchase.^17^ We assumed that the topic of food quality from a DIY perspective would have been well received by scientists both internally the JRC (not least, in the light of the recent enthusiasm around Citizen Science approaches^18^) and in the wider DIY community (Table 4)

**Table 4:** Auto-ethnography stages

#	Experiment stage	Main objective
1	Feasibility study Identification of the actors and preliminary technical hypothesis	A feasibility study on the application if DIY method for food quality assessment Selection of pool of actors both internally the JRC and in the DIY community
2	Identification of possible areas of application	Review of scientific literature on a selection of applicative cases (detection of nitrites/nitrates through semi quantitative colour-based spectrophotometry, UV and IR Arduino-based spectrophotometry)
3	Hands on testing	Prototyping of different practical possibilities. Fig. 1 shows an. Arduino based kit for the detection of nitrite/nitrate in food samples. A TCS3200 colour sensor in a controlled dark room is employed to read commercially available test strips.^s^ In a second stage we investigated the reproducibility of these strips through DIY means, although we did not proceed with the actual realization.
4	Collection and analysis of results and actors ‘interactions	Data collection (oral and written communications with actors, online and offline material)

^a^See e.g. http://www.lamotte.com/en/food-beverage/test-strips/2996.html. Retrieved 12/10/2018

.

**Fig. 1:**
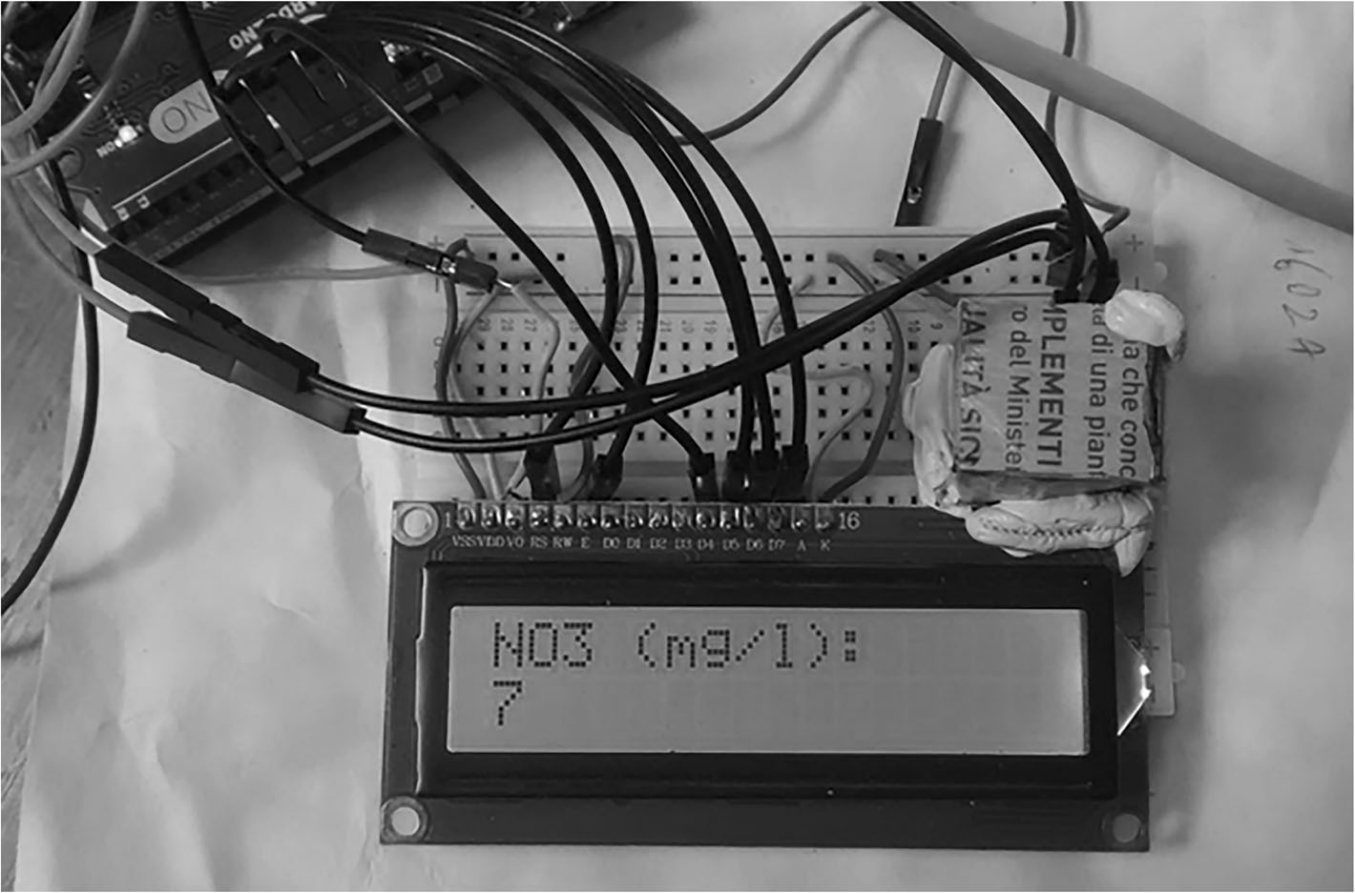
“Arduino based prototype for automatic test strip reading”. A TCS3200 colour sensor in a controlled dark room is employed to read commercially available test strips. Copyright of the author

We decided to conduct our experiment as follows: (Fig. 1)

Data analysis consisted of transcribed annotations, as well as blog activity and personal written and oral communications. Data were collected in transcripts and treated with QDA® miner software which allows categorization and coding as well as intelligent retrieving of content. We created several entries with information on the actors, spaces, personal communications and interactions object of our study and collected during both of the above described research phases. These include geographical location, date, technical characteristics, organizational aspects, legal context and personal observations of the visited spaces, as well as demographic and background details, activities, formal commitments and transcripts of communications or statements of the actors we interacted with. We analysed recurring themes along the principles of grounded theory as displayed in Charmaz (2006). We grouped and named the descriptive codes via software, next to the main topics of the treated excerpts.

This process drew the basis for the production of Tables 5 and 6.

## Analysis

The question guiding our inquiry into DIY science is: what are actors’ actions and commitments that describe the social worlds operating in the context of DIY science?

Strauss (1978) argues that social worlds and arenas refer centrally to *universes of discourse* (p. 121) and it is precisely after the articulation of discourse that we posed our attention when analysing our research data: how do actors articulate views on DIY science and on the actions of their worlds? How are affiliations, memberships and relations built and sustained within groups, associations, communities and organizations?

Table 5 presents the actors of our study and their actions in DIY science premises:

**Table 5:** Actors and actions

Actor	Phase	Context(s)	Actions
1	1	Hacker movement, Citizen Science	Development and study of new forms of public participation in research - Use of open source software for ‘gamification’ as a strategy to engage citizens in projects around local challenges (e.g. disease mapping) – Training and public events participation with a focus on open source and citizen science
2	1	Entrepreneurship	Launch of a start-up aimed at assessing and distributing ‘value’ in a specific ecosystem according to principles of share, collaboration and open source – Strong participation to discussions, panels, and public talks on the themes of collaboration
3	1	Private consultancy	Private consultancy in the field of automation, artificial intelligence, the future of manufacturing, the Maker Movement – Editing of reports and publication on these themes
4	1	Maker Movement, Academia	Direction of a Fablab located inside the faculty of design of a university – organisation of European events on the Maker Movement – Assistance to students at various prototyping phases of their projects and research
5	1	Maker Movement	Direction of a makerspace, organization of more projects (even institutionally funded) on open source applied to the contexts of fabrication, design, health – Technical workshop organisation
6	1	Maker Movement, Entrepreneurship	Direction of a Fablab designed as a provider of open source technology to local enterprises that are registered to the association – Active participation to workshops and seminars - Drafting of open calls for public funding
7	1	Bio Hacking	Management of many ventures in the field of decentralized networks and innovation. Self-experimentation with human enhancement techniques and biohacking – Online sharing of outcomes and data
8	2	DIY biology	Participation to hackerspace’s activity in the field of DIYbio with major interest on personal projects development – Events organisation in Academia with the scope of informing scientists about the DIY bio community - Online sharing of outcomes and data
9	2	Institutional Research	Research in biology and food contaminants in a public research centre – Personal intellectual interest in DIY applications and emerging technologies – Scientific support to policy making
10	2	DIY biology	Coordination of online community concerned with the development of software and hardware for a variety of projects put forward by the community (e.g. air quality sensing, optics and imaging, mobile spectrometry)
11	2	Institutional Research	Conduction of a reference laboratory for food analysis located inside a public research centre - Private experimentation with DIY technologies

After analysing the actions of the actors selected from our field research, we were able to draft a tentative pool of the SWs of DIY science. We unpacked some of these SWs according to the descriptive guidelines of Clarke (2003, p.115). A summary is presented in Table 6, which we subsequently used as a basis for possible mappings of DIY science. For each SW we collected the main commitments as they have been articulated by the actors in Table 5, accompanied by the diversity of descriptions of the worlds, the actions, technologies taking places within different sites and, possibly, organizations.

**Table 6:** Social worlds

SW	Commitments	Self-Descriptions of the SW (readapted from actors’phrasing)	Relations with other SWs	Actions and *technologies*	Site of actions and/or organizations
#1 Maker Movement	-Democratization of productive processes -Alternative learning -Citizen empowerment	“A culture based on a different set of values” “The members of Fablabs and makerspaces” “A learning opportunity and educational model” “Everyone using a 3d printer”	-Citizen Science -Entrepreneurship -DIY bio	-Sharing of spaces, resources and technology -Art/Science practices -Online support -Education -Participation to events -*3D print, microcontrollers,* etc.	-Fablabs, makerspaces -Resource websites -Community events -Schools -Museums
#2 Hacker Movement	-Democratization of software -Sharing of information on technical and societal issues (privacy, surveillance, activism)	“Similar to the Maker Movement but totally non-institutionalized. Fablabs are not the hacker movement” “The collective community operating online for open source software and privacy protection” “Online activism, sometimes in a grey area” “A cultural change”	-Maker Movement -Citizen Science	-Programming -Online support and activism *-Open software and hardware*	-Virtual spaces -Dedicated events -Hackerspaces (for educational purposes)
#3 Bio Hacking	-Enhancement of self being -Individual and collective wellbeing -Cybernetics	“Playing the doctor of yourself” “Experimenting with the human body for psychophysical enhancement” “DIY clinical research”	-DIY bio	-Body modification -Neurohacking -Drug development -Online support *-Nootropics* *-Biometric data*	-Online communities -Dedicated events
#4 DIY Bio	-Sharing of knowledge and resources for research in biology -Citizen science support	“Conducting experiment out of the lab for specific purposes and even for fun” “Traditional biology research carried out even by non-professionals” “DIY Bio and biohacking are often mistaken”	-Bio Hacking -Citizen Science -Maker Movement -Entrepreneurship	-Experiments and trials set up -Formal Research -Online support -Art/Science practices -*Printed biosensors* *-Genome editing techniques*	-Hackerspaces -Private spaces -Museums -Dedicated events
#5 Citizen Science	-Citizen participation to science activities -Local knowledge -Local problem solving -Public consultation	“Bottom up scientific initiatives to tackle local or community problems” “An opportunity to integrate scientific knowledge in certain circumstances” “The top down engagement of citizens in science”	-Maker Movement -DIY bio -Hacker Movement	-Sharing of spaces, resources and technology -Community support -Interfacing with policy and the academia -*Different technologies and apps*	-Makerspaces and Fablabs -Local spaces -Online platforms
#6 Entre- preneurship	-Production of innovation -Starting businesses -Alternative business models	“Capitalization of DIY approaches for business purposes” “Expansion of access to services and products according to open source” “Reconsideration of labour in a more sustainable way”	-DIY bio -Maker Movement -Hacker Movement	-Use of new technologies and emerging communities for private business *-Different technologies*	-Makerspaces, Fablabs and hackerspaces -Dedicated events

After identifying the main constitutive SWs of DIY science, we are ready to aggregate them in arenas. Clarke (2003) offers a number of indications to be checked against when taking the arena mind-set (p.124). In Table 7, examples of these criteria can be found based on the conceptualisation of DIY science as a potential arena of interest. These criteria are generally valid for thinking about any social arenas.

**Table 7:** Criteria to brainstorm about arenas (See Clarke, 2003 p. 124)

• Around the chosen arena, no major social worlds have appeared	
Our literature review shows multiple perspectives over the DIY science phenomenon, as well as its multi-faceted intrinsic nature. DIY science contains different actors and collectives that respond to different cultures and practices. No actors at both research phases stressed the prevalence of any SW against others.	
• Historically, we have a sense of the changes that have interested the arena at stake	
Many aspects have characterized the latest developments of various instances of DIY science (e.g. new technologies such as 3d printing), which can therefore be considered a further development of an already existing phenomenon	
• Social worlds can in turn be deconstructed in arenas	
We think this could be the case of DIY biology or other SWs within DIY science (e.g. IT hacking, the Maker Movement, etc.). We explore this possibility by offering two distinct possibilities of mapping.	

### Mapping DIY science

The reflection on our situational elements can result in different possibilities of mapping the arenas of DIY science.

One possibility might originate from selecting “DIY science” as the arena *containing* the totality of the SWs described in Table 6.

However, along the third criterion in Table 7, nothing prevents us from deconstructing single SWs into arenas on their own. In all cases, boundaries between arenas are to be considered flexible and porous (from which, they are represented by dotted lines).

The analysis of the self-descriptions of the SWs of our study suggests that some SWs are typically conceptualised in different ways by different actors (see Table 6). We took the case of the Maker Movement, around which there are abundant viewpoints and stances, both in our empirical material, as well as on the Internet.

We investigated how different conceptualization of the Maker Movement can lead us to different mappings of DIY science: Map 1 (called “the Maker Movement arena”) and Map 2 (called “The DIY science arena”) follow from differences that have emerged in our first field research phase in the discourse about the Maker Movement.

In a first possibility, the Maker Movement alludes to the “DIY empowerment” paradigm of everyone that operates creative skills to design and make objects, as well as applies peer-to-peer based learning to solve problems. From such view, makers can also be individually active, since no particular reference is made to their operational centres, neither to their specifically employed technologies. “Making” is depicted as something that has always existed, as it does not depend on the technological aspect, rather on a cultural mind-set (see e.g. Burke, 2014). In other words, someone fiddling around broken electronics in their garage could be considered a maker by all means. Surely akin to the counterculture of the 60s an70s, this conceptualization of the Maker Movement sometimes recalls a moral critique to the passivity of consumerism societies (see e.g. Smith, 2014) This is mirrored, for instance, in the words of Actor 5 of the first phase (see Table 5), who stated that *“makers aren’t just people who create objects; it’s everyone experimenting with new ways of living”*.

In a second case, the conceptualisation of Maker Movement is instead seen restricted to its more technical and institutionalised aspects. Makers are more precisely identified with specific actors (designers, artists, IT developers, urban planners, etc.) making use of specific technologies (e.g. 3d printing, Arduino, etc.) and typically active in a well-defined community setting (e.g. Makerspaces, Fablabs). This meaning of the Maker Movement is reflected by quotes such as “*there are two challenges that makers face as they begin the creation process: a physical place in which to build, and a “coach” of sorts to help them through what can be a complex process*”.^19^ Also, this more specific understanding of the Maker Movement is characterised by the *novelty* with which makers are expected to *deliver* their contributions. The European Maker Week is an initiative promoted by European Commission that aims to attract European citizens to the “Maker world”. In its website it is stated that “*the Maker Movement is the name given to the increasing number of people coming from different backgrounds, who are employing do-it-yourself (DIY) and do-it-with-other (DIWO) techniques and processes to develop unique technologies and products as well innovative solutions”*^20^

A private consultant (Actor 3) from phase 1 discussed makers as “*those who are affiliated to either a makerspace or a Fablab*”, while a Fablab director (Actor 6) recalled that “*typically when you plan initiatives, you are targeting a specific community that has its reference events in fairs and its location in the Fablab network*”.

With this example we see howthe discourse about the Maker Movement is thus performative in generating different options of mapping social worlds and arenas, and thus indirectly, conceptualizing DIY science. We tried to exemplify this through Map 1 and Map 2, which were obtained adopting the graphical standards of SW/A mapping presented in Clarke (2003). According to Map 1 not all makers are necessarily DIY scientists (as people fiddling around broken electronics are also makers), while all DIY scientists are also makers (as they adopt a DIY paradigm). This is no longer the case in Map 2, where the Maker Movement is made to coincide with the institution of makerspaces, Fablabs and community spaces, as we might assume that some DIY scientists are active outside specific communities of practice or networks, e.g. individually in their private labs.

**Fig. 2:**
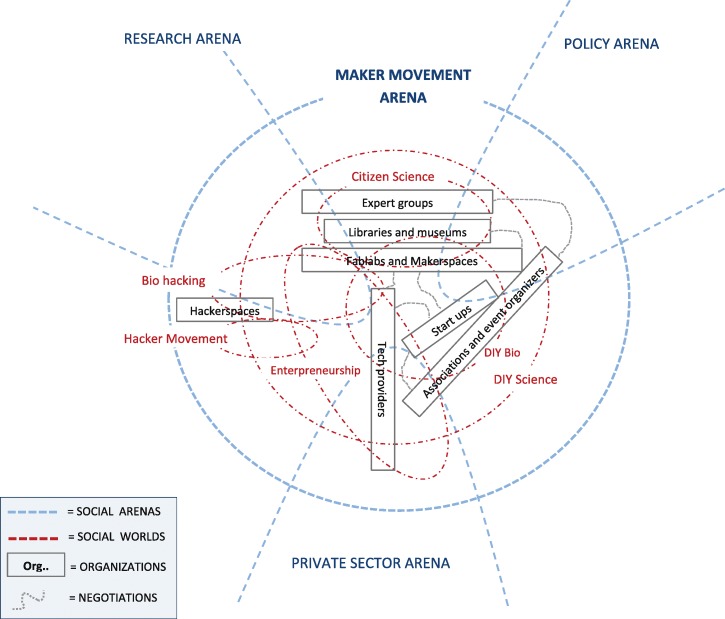
“Map 1- The Maker Movement Arena” Map 1 is obtained from conceptualizing the Maker Movement as a social arena. The constitutive elements in the figure have been produced along the working suggestion in Clarke (2003)

**Fig. 3:**
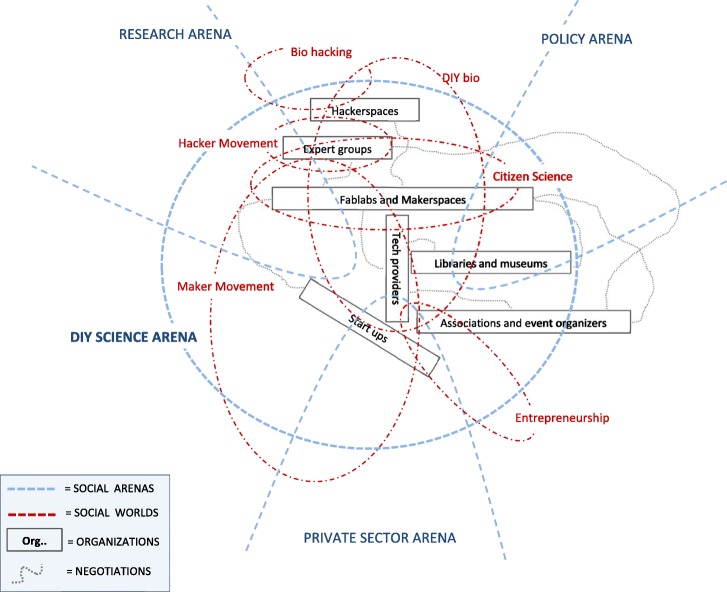
“Map 2- The DIY Science Arena” Map 2 is obtained from conceptualizing the DIY science as a social arena. The constitutive elements in the figure have been produced along the working suggestions in Clarke (2003)

Although we know that there are no close ended definitions of the Maker Movement, differences (even marginal) in its conceptualisation can result in misconceptions when objects of discourse such as “makers”, “makerspaces”, or “DIY scientists” are targeted. A recent article published on the World Economic Forum and entitled “Why makerspaces could be the secret to making smart cities smart”^21^ praise the Maker Movement for its capacity to become a key player in urban transformation over the coming decades, specifically in economic and environmental terms. Yet, although frequently recalled, the identity of makers is not really discussed, somehow leaving it to the idea of tech enthusiasts “coming up with better, smarter, more efficient solutions for producing goods and delivering services”. Such view seems to resonate with how modern framings of buzzwords such as “sustainability” are functional to a certain understanding of innovation (Benessia & Funtowicz, 2015), whereby society continuously stands in a position for the better, the smarter, and the more efficient. Are we certain that makers share a similar idea of what needs to be better, smarter, and more efficient? (Figs. 2 and 3)

### Discussion

Situational maps are a powerful tool to explore the social situatedness of complex phenomena such as DIY science. We have shown that the analysis of discourse circulating on DIY science can lead to different possibilities of conceptualizing its situational elements. Why are Map 1 and Map 2 both equally plausible and legitimate? What are the consequences of such potential plurality?

Clarke (2003) asserts that “social worlds are universes of discourse in arenas constituted and maintained through discourse” (p.114). From an analyst standpoint, discourse thus becomes performative in creating certain social worlds and arenas against others. Consequently, notions of particular social worlds and arenas directly derive from the articulation of discourse: discussing DIY science indirectly co-creates its situatedness, which is not free from risks of blind assumptions, unmotivated statements and factual misconceptions.

In an article on the development of discourse about ‘buzzwords’ and ‘fuzzwords’, Cornwall (2007) stressed how “words make worlds”, alluding to how language itself animates and justifies intervention with promises of what is deemed possible.

Policy spheres are rich in examples of use of specific notions of DIY science that not always reflect the views of actors within the DIY community. A summary of a held session entitled “European Stakeholder Round Table on Citizen, DIY Science and Responsible Research and Innovation” carried out by the European Citizen Science Association (ECSA) states “funding, recognition of legitimacy and better links within the research community” as the “immediate problems” that DIY and citizen science groups are facing.^22^ Such understanding is not quite shared by some of the actors encountered during our auto-ethnography. First, the community of DIY biologists, with its online reference portals, lacks of a clear and unified objective, due to the high diversity in the practitioners’ expertise and aspirations. In the field of spectrometry, for instance, one shared goal is to specifically maximize and improve inexpensive techniques in order to allow the flourishing of a collaborative network completely independent from institutional funding and regulation. Second, different visions underpin interest in food quality assessment. For example, our project on the DIY screening of nitrates/nitrites concentrates in food samples was well received both internally our research centre, by our colleagues in the field, as well as by the wider DIY community. However, we noticed how professional scientists have tried to accommodate our proposal to develop a DIY sensor somehow including it in the list of their institutional deliverables, aiming to integrate their already existing work with a DIY approach. While pre-fixed objectives seem to be the norm and driving force in formal institutions, practitioners not institutionally affiliated not always consider that what applies to institutional research has to be relevant in DIY science premises. Indeed, independent DIY scientists typically seemed keener to explore the topic independently from the supposed scientific importance and implications. Such issue of generalization was rendered visible by a lab technician employed in our research centre: while he could not find much space for accommodating our project into his professional activity, he showed remarkable interest personally as a private citizen, to such an extent that he asked to be involved in the project outside his working time.

Professional identity plays a key role among DIY scientists. A survey published by the Woodrow Wilson International Centre for Scholars 23, showed that in 2013 approximately a third of participants of the DIY Bio community biology held PhDs and had positions in a government-funded or privately-owned research institute. The fact that ours was an institutional project caused mixed reactions both internally and outside the research centre. Typically practitioners foresee participation with different perspectives according to whether DIY science projects are presented in a professional environment rather than in informal premises, such as a garage or a local makerspace.

DIY science is frequently constrained by practical and technical limits. The development of our sensor revealed that infra-red spectrometry is hard, if not impossible, to be conducted without the advantages of professional laboratories. Conversely, while this was interpreted as a point of failure of the DIY approach by colleagues in mainstream research, the online community of amateur biologists considered it as a positive achievement, whereby the added value of the DIY paradigm resides in the entire process, made of freedom of exploration, as well as pure entertainment.

It comes straightforward that different attitudes towards DIY science would translate into different possibilities of mapping the very same actors: is he a laboratory technician, a free time DIY biologists, or both?

Every account on DIY science should carefully take into consideration what particular notions of DIY science are selected by policy, media and the community and for which purpose. Whenever the discourse culminates with direct or indirect conception of close ended notions, attention must be paid to their possible implications. In designing funding campaigns aimed at supporting economically the Maker Movement, for instance, a policymaker could either rely on Map 1 or Map 2 (or even others). If one assumes Map 1, additional social worlds and their actors (e.g. citizen scientists, or start-ups) should be included in the coverage of the investment, as they are part of the broader Maker Movement arena. According Map 2, these same social worlds might end up being excluded or only partly targeted (e.g., “not all citizen scientists are makers”).

Different notions of DIY science and its social worlds therefore lead to different situational options, which in turn can have implications in terms of potential exclusion of relevant actors, missed opportunities, and even instrumentalisation for hidden agendas.

DIY science is bound to remain an unstable etity, perhaps even an “essentially contested concept” (drawing from Gallie, 1956), similarly to what recently suggested by Korhonen, Nuur, Feldmann, & Birkie (2018) about the notion of “circular economy”.

Every use of DIY science in public discourse should therefore be accompanied with detailed information about the situational elements it entails, and a careful contextualization of its social worlds and actors. Only in such a way can we try to avoid the risk of appropriation and instrumentalisation of DIY science without consideration of the complex multitude of interests, imaginations, expectations and assumptions it embeds.

## Conclusions

With the present study we demonstrated that no definition of DIY science taken alone is meaningful or fully comprehensive of the variety with which DIY science expressions have emerged and shall emerge in the future. DIY science becomes “objectified” as an entity of discourse as soon as it gets articulated *through* discourse. By applying the theory of social worlds/arenas we were able to show how more conceptualisations and interrelations of DIY science are possible and legitimate at the same time. This has important ontological consequences as soon as DIY science becomes a topic of discussion in research, policy and governance. Are, for instance, individually active so-called “biohackers” also DIY scientists? Taking “DIY science” as unique and stable inevitably leads to major reductionisms that can range from epistemic exclusions and indirect facilitation of some worlds (groups, communities, organization, institutions), to the misrepresentation, myth and hype about the real scientific implications of DIY science.

In relation to such reductionisms, Clarke (2003) says that “because social worlds/arenas attempt to represent most if not all of the major social worlds […] the analyst grants greater power to the less powerful worlds (p.124)”. With our auto-ethnography, we exemplified how in the case of DIY Bio, some communities tend to be moved by different concerns for action than those of institutional worlds, although the visibility and outreach of their practices is set on a totally different scale.

## Abbreviations

CRISPR: Clustered Regularly Interspaced Short Palindromic Repeats
DIY Bio: Do-It-Yourself Biology
DIY: Do-It-Yourself
ECSA: European Citizen Science Association
SW/A: Social worlds/arenas

## Footnotes

1 2015 survey-based study by the Pew Research Centre and the American Association for the Advancement of Science (AAAS) highlighted how in the United States a considerable opinion gap still exists between the general public and scientists on a wide range of science and technology topics, for instance, climate change and human activity or favour use of animals in research. http://www.pewinternet.org/interactives/public-scientists-opinion-gap/. Retrieved 30/05/2018.

2 https://www.theguardian.com/science/2015/dec/04/human-gene-editing-is-a-social-and-political-matter-not-just-a-scientific-one. Retrieved 16/04/2018.

3 See e.g. an issue of the Time published in 1954 entitled “Do-It-Yourself: the New Billion Dollar Hobby”. http://content.time.com/time/covers/0,16,641,19,540,802,00.html Retrieved 11/10/2018

4 http://setiathome.berkeley.edu/sah_about.php Retrieved 10/10/2018

5 http://www.zdnet.com/article/14-million-raspberry-pis-sold-10-million-made-in-the-uk/. Retrieved 10/02/2018

6 The query was set to match the strings “DIY science” or “do-it-yourself science” either in the title, abstract, or keywords. All articles have then been manually scrutinized for pertinence. Query specifications: (ALL (“do-it-yourself science”) OR ALL (“DIY science”)) From www.scopus.com. Retrieved 10/04/2017

7 When performing this literature review, we were expecting a greater deal of papers concerned with the education and learning aspects of DIY science. Around the topic of maker movement, education and learning occupy the larger share of scholarly contributions.

8 https://www.theguardian.com/higher-education-network/blog/2014/jun/16/diy-labs-exciting-alternative-university-science-research. Retrieved 22/02/2018. The Guardian launched a dedicated blog called “Academics Anonymous”, devoted to give voice to scientists and let them “tell it as it is”.

9 See e.g. https://ecsa.citizen-science.net. Retrieved 02/03/2018. The Horizon 2020 framework program alludes to promote “citizens’ interests and capacities for science allowing them to actively participate in various scientific activities (e.g. exhibitions and science cafés, grass roots “Do It Yourself” (DIY) creative re-use communities, on-line mechanisms for knowledge-based policy advice)” (European Commission, 2017, p. 21).

10 https://www.theguardian.com/higher-education-network/blog/2014/jun/16/diy-labs-exciting-alternative-university-science-research. Retrieved 12/04/2018.

11 https://en.unesco.org/news/unesco-panel-experts-calls-ban-editing-human-dna-avoid-unethical-tampering-hereditary-traits. Retrieved 06/03/18.

12 https://waag.org/en/blog/genome-editing-symposium-knaw. Retrieved 06/03/18.

13 Online Etymology Dictionary: https://www.etymonline.com/word/situation. Retrieved 09/03/18.

14 For details about what is a Fablab please refer to http://www.fablabni.com/what-fablab.html. Retrieved 13/03/18.

15 Joint Research Centre of the European Commission (JRC). See https://ec.europa.eu/jrc/en/research-topic/food-and-feed-safety.

16 See e.g. http://nypost.com/2016/07/10/the-truth-behind-how-were-scammed-into-eating-phony-food/. Retrieved 16/04/2018. https://www.nytimes.com/2014/09/19/opinion/taiwans-gutter-oil-scandal.html. http://www.bbc.com/news/world-asia-29249180. Retrieved 17/03/18.

17 Joint Research Centre of the European Commission (JRC). The report is based on the outcomes of media analysis (Europe media monitor) and internet monitoring tools such as the Rapid Alert System for Food and Feed (RASFF) portal. Retrieved 28/03/2018 See report at https://ec.europa.eu/jrc/sites/jrcsh/files/jrc-food-fraud-summary-september-2016.pdf Retrieved 28/03/2018

18 Joint Research Centre of the European Commission (JRC). See e.g. http://digitalearthlab.jrc.ec.europa.eu/activities/citizen-science-platform-%E2%80%93-tool-extend-evidence-base-policy/57787. Retrieved 28/03/2018

19 Extracted from http://www.hult.edu/blog/an-overview-of-the-maker-movement-what-is-it-and-why-now-part-1/ Retrieved 12/04/2018.

20 https://europeanmakerweek.eu/about-us/. Retrieved 12/04/2018.

21 https://www.weforum.org/agenda/2018/03/makerspaces-smart-sustainable-cities-thomas-ermacora/. Retrieved 12/04/2018.

22 https://ecsa.citizen-science.net/blog/european-stakeholder-round-table-citizen-diy-science-and-responsible-research-and-innovation. Retrieved 04/05/2018.

23 https://www.wilsoncenter.org/event/myths-realities-the-diybio-movement. Retrieved 16/05/2018.

## References

[CR1] Alper M (2013). Making space in the makerspace: building a mixed-ability maker culture. Interaction design and children conference.

[CR2] Andersen NB (2014). “Dioxins are the easiest topic to mention”: resident activists’ construction of knowledge about low-level exposure to toxic chemicals. Public Underst Sci.

[CR3] Austen K. Out of the lab and onto the streets. New Scientist. 2013; 10.1016/S0262-4079(13)61628-0.

[CR4] Bardaji R, Sánchez AM, Simon C, Wernand MR, Piera J. Estimating the underwater diffuse attenuation coefficient with a low-cost instrument: the KdUINO DIY buoy. Sensors (Switzerland). 2016;16(3) 10.3390/s16030373. 10.3390/s16030373 PMC4813948 26999132

[CR5] Benessia A, Funtowicz S (2015). Sustainability and techno-science: what do we want to sustain and for whom?. Int J Sustainable Development J Sustainable Development.

[CR6] Berditchevskaia, A., Regalado, C., & Duin, S. van. (2017). The changing face of expertise and the need for knowledge transfer. J Sci Commun, 16(4):1–8.

[CR7] Brown, J. (2017). Citizen Science: if you want something doing, do it yourself. Chapter in Biochemist. Magazine of the Biomedical Society. 39(5):42–45.

[CR8] Brown J, Crocamo JT, Bielskas A, Ransom E, Vanti WB, Wilfong K (2017). Evolving skills for emerging technologies: a collaborative approach. Library Hi Tech.

[CR9] Buechley L, Perner-Wilson H (2012). Crafting technology: reimagining the processes, materials, and Cultures of Electronics. ACM Transactions on Computer-Human Interaction.

[CR10] Burke J (2014). Making Sense: Can Makerspaces Work in Academic Libraries?.

[CR11] Busch JA, Bardaji R, Ceccaroni L, Friedrichs A, Piera J, Simon C, Thijsse P, Wernand M, van der Woerd HJ, Zielinski O. Citizen bio-optical observations from coast- and ocean and their compatibility with ocean colour satellite measurements. Remote Sens. 2016;8(11) 10.3390/rs8110879.

[CR12] Charmaz, K. (2006). Constructing grounded theory: a practical guide through qualitative analysis. Book (Vol. 10). 10.1016/j.lisr.2007.11.003.

[CR13] Chen Y, Wu C (2017). The hot spot transformation in the research evolution of maker. Scientometrics.

[CR14] Chen Y, Wu C (2017). The hot spot transformation in the research evolution of maker. Scientometrics.

[CR15] Clarke AE (2003). Situational analyses: grounded theory mapping after the postmodern turn. Symb Interact.

[CR16] Cooper CB, Shirk JL, Zuckerberg B (2014). The invisible prevalence of citizen science in global research: migratory birds and climate change. PLoS One.

[CR17] Cornwall A (2007). Buzzwords and fuzzwords: deconstructing development discourse. Dev Pract.

[CR18] Cressey D. PhDs leave the ivory tower. Nature. 2012; 10.1038/484020a. 10.1038/484020a 22481335

[CR19] Cressey D (2017). The DIY electronics transforming research. Nature.

[CR20] Dance A (2017). Solo scientist. Nature.

[CR21] Davies SR, Tybjerg K, Whiteley L, Söderqvist T (2015). Co-curation as hacking: biohackers in Copenhagen’s medical Museion. Curator.

[CR22] Day, R. J. F. (2005). Gramsci is dead. Pluto Press. Retrieved from http://www.jstor.org/stable/j.ctt18fs4xw

[CR23] Delgado A (2013). DIYbio: making things and making futures. Futures.

[CR24] Duncombe S (1997). Notes from underground: Zines and the politics of alternative culture.

[CR25] Eggleson, K. (2014). Transatlantic divergences in citizen science ethics—comparative analysis of the DIYbio code of ethics drafts of 2011. NanoEthics, 8(2), 187–192. http://doi.org/10.1007/s11569-014-0197-7

[CR26] European Commission. (2017). Horizon 2020 Work Programme 2014–2015: 16. Science with and for Society. *European Commission*, (October). Retrieved from http://ec.europa.eu/research/participants/data/ref/h2020/wp/2014_2015/main/h2020-wp1415-swfs_en.pdf

[CR27] Fortunati L, Esposito A, Ferrin G, Viel M (2014). Approaching social robots through playfulness and doing-it-yourself: children in action. Cogn Comput.

[CR28] Fourie I, Meyer A (2015). What to make of makerspaces: tools and DIY only or is there an interconnected information resources space?. Library Hi Tech.

[CR29] Fritsch M, Krabel S (2012). Ready to leave the ivory tower?: academic scientists’ appeal to work in the private sector. J Technol Transfer.

[CR30] Gallie WB (1956). Essentially contested concepts. Proc Aristot Soc.

[CR31] Garbarino J, Mason CE (2016). The power of engaging citizen scientists for scientific Progress. J Microbiol Biol Educ.

[CR32] Garrety K (1998). Science, policy, and controversy in the cholesterol arena. Symb Interact.

[CR33] Gauchat G (2012). Politicization of science in the public sphere. Am Sociol Rev.

[CR34] Gelber SM (1999). Hobbies : leisure and the culture of work in America/Steven M. Gelber.

[CR35] Haklay M, Mazumdar S, Wardlaw J, Mathieu P-P, Aubrecht C (2018). Citizen Science for Observing and Understanding the Earth BT. Earth Observation Open Science and Innovation.

[CR36] James M (2015). Synthetic biology and morality: artificial life and the bounds of nature. The New Bioethics.

[CR37] Karlberg K. The work of genetic care providers: Managing uncertainty and ambiguity. In: Health Care Providers, Institutions, and Patients: Changing Patterns of Care Provision and Care Delivery (Vol. 17, pp. 81–97): Emerald Group Publishing Limited; 2000. 10.1016/S0275-4959(00)80040-X.

[CR38] Kelty CM (2010). Outlaw, hackers, victorian amateurs: diagnosing public participation in the life sciences today. J Sci Commun.

[CR39] Korhonen J, Nuur C, Feldmann A, Birkie SE (2018). Circular economy as an essentially contested concept. J Clean Prod.

[CR40] Kumar, P., Morawska, L., Martani, C., Biskos, G., Neophytou, M., Di Sabatino, S., … Britter, R. (2015). The rise of low-cost sensing for managing air pollution in cities. Environ Int 10.1016/j.envint.2014.11.019. 10.1016/j.envint.2014.11.019 25483836

[CR41] Kuznetsov, S., & Paulos, E. (2010). Rise of the expert amateur : DIY projects , communities , and cultures. Proceedings of the 6th Nordic Conference on Human-Computer Interaction: Extending Boundaries, (Figure 1), 295–304. 10.1145/1868914.1868950

[CR42] Lamore B (2016). Fan cart: the next generation. Phys Teach.

[CR43] Landrain T, Meyer M, Perez AM, Sussan R (2013). Do-it-yourself biology: challenges and promises for an open science and technology movement. Syst Synth Biol.

[CR44] Lave R (2012). Neoliberalism and the production of environmental knowledge. Environ Soc.

[CR45] Lehr C, Rauneker P, Fahle M, Hohenbrink TL, Böttcher S, Natkhin M (2017). Communicating landscape hydrology — the water cycle in a box. Hydrol Process.

[CR46] Lin YW (2011). A qualitative enquiry into OpenStreetMap making. New Review of Hypermedia and Multimedia.

[CR47] Lindtner, S., Hertz, G. D., & Dourish, P. (2014). Emerging sites of HCI innovation. Proceedings of the 32nd Annual ACM Conference on Human Factors in Computing Systems. Retrieved from 10.1145/2556288.2557132

[CR48] Lindtner S, Lin C (2017). Making and its promises. CoDesign.

[CR49] Lowndes S (2016). The DIY Movement in Art, Music and Publishing.

[CR50] Menninger WC (1942). Psychological aspects of hobbies. Am J Psychiatr.

[CR51] Mereu FJ, Villarroel J. Visions project K.1: DIY 3-D interactive videohologram device. Int J Arts Technol. 2014;7(4).

[CR52] Morzy M (2015). ICT services for open and citizen science. World Wide Web.

[CR53] Nascimento S, Guimarães Pereira Â, Ghezzi A. From citizen science to do it yourself science; 2014. 10.2788/12246.

[CR54] Neilson SJ, Kai J, McArthur C, Greenfield S (2013). Using social worlds theory to explore influences on community nurses’ experiences of providing out of hours paediatric palliative care. J Res Nurs.

[CR55] Newman G, Wiggins A, Crall A, Graham E, Newman S, Crowston K. The future of citizen science: emerging technologies and shifting paradigms. Front Ecol Environ. 2012; 10.1890/110294.

[CR56] Nguyen J (2016). Make magazine and the social reproduction of DIY science and technology. Cultural Politics.

[CR57] Rennie LJ, Evans RS, Mayne FE, Rennie SJ (2010). Factors affecting the use and outcomes of interactive science exhibits in community settings. Visitor Studies.

[CR58] Richards J (2016). Shifting gender in electronic music: DIY and maker communities. Contemp Music Rev.

[CR59] Salin O, Pesso K (2017). Open minds, open spaces: mind-set changes during urban walking. Space and Culture.

[CR60] Seyfried G, Pei L, Schmidt M (2014). European do-it-yourself (DIY) biology: beyond the hope, hype and horror. BioEssays.

[CR61] Sleator RD (2016). Diy biology-hacking goes viral!. Sci Prog.

[CR62] Sleator RD (2016). Synthetic biology: from mainstream to counterculture. Arch Microbiol.

[CR63] Smith CD (2014). Handymen, hippies and healing: social transformation through the DIY movement (1940s to 1970s) in north america. Architectural Histories.

[CR64] Strauss A (1978). A social world perspective. Studies in Symbolic Interaction.

[CR65] Strickland E (2014). Brain hacking: self-experimenters are zapping their heads. IEEE Spectr.

[CR66] Tofel-Grehl C, Fields D, Searle K, Maahs-Fladung C, Feldon D, Gu G, Sun C (2017). Electrifying engagement in middle school science class: improving student interest through E-textiles. J Sci Educ Technol.

[CR67] Trumbull, D. J., Bonney, R., Bascom, D., & Cabral, A. (2000). Thinking scientifically during participation in a citizen-science project. Sci Educ, 84(2), 265–275. 10.1002/(SICI)1098-237X(200003)84:2<265::AID-SCE7>3.0.CO;2-5.

[CR68] Van Rijswijk W, Frewer LJ (2012). Consumer needs and requirements for food and ingredient traceability information. Int J Consum Stud.

[CR69] Vandevelde C, Wyffels F, Ciocci MC, Vanderborght B, Saldien J (2016). Design and evaluation of a DIY construction system for educational robot kits. Int J Technol Des Educ.

[CR70] Vasconcelos, A. (2007). The use of grounded theory and of arenas/social worlds theory in discourse studies: a case study on the discursive adaptation of Inf Syst, 5. Electronic Journal of Business Research Methods Volume 5 Issue 2 2007 (125–136)

[CR71] Wan S, Wu B, Chen N (2007). Application of program generation technology in solving heat and flow problems. J Therm Sci.

[CR72] Wexler A. The social context of “do-it-yourself” brain stimulation: Neurohackers, biohackers, and Lifehackers. Front Hum Neurosci. 2017;11 10.3389/fnhum.2017.00224. 10.3389/fnhum.2017.00224 PMC5423946 28539877

[CR73] Wylie SA, Jalbert K, Dosemagen S, Ratto M (2014). Institutions for civic Technoscience: how critical making is transforming environmental research. Information Society.

[CR74] Wynne B (2007). Public participation in science and technology: performing and obscuring a political–conceptual category mistake. East Asian Science, Technology and Society: An International Journal.

[CR75] Yamoah FA, Yewson DE (2014). Assessing supermarket food shopper reaction to horsemeat scandal in the UK. Int Rev Manag Mark.

[CR76] Yang B, Patsavas MC, Byrne RH, Ma J (2014). Seawater pH measurements in the field: a DIY photometer with 0.01 unit pH accuracy. Mar Chem.

